# Barriers to participation in biosampling-based translational research: A cross-sectional survey of Canadian critical care researchers

**DOI:** 10.1371/journal.pone.0303304

**Published:** 2024-05-17

**Authors:** Erblin Cani, Jennifer L. Y. Tsang, Alexandra Binnie, Claudia C. dos Santos, Robert Fowler, Francois Lamontagne, Sangeeta Mehta, Patricia C. Liaw, on behalf of the Canadian Critical Care Translational Biology Group (CCCTBG), and the Canadian Critical Care Trials Group (CCCTG)

**Affiliations:** 1 Department of Medical Sciences, McMaster University, Hamilton, ON, Canada; 2 Thrombosis and Atherosclerosis Research Institute, Hamilton, ON, Canada; 3 Department of Medicine, McMaster University, Hamilton, ON, Canada; 4 Niagara Regional Campus, Michael G. DeGroote School of Medicine, McMaster University, St. Catharines, Ontario, Canada; 5 Niagara Health, St. Catharines, Ontario, Canada; 6 Intensive Care Department, William Osler Health System, Etobicoke, ON, Canada; 7 Keenan Research Center for Biomedical Research, Unity Health Toronto, Toronto, ON, Canada; 8 Interdepartmental Division of Critical Care and Institute of Medical Sciences, University of Toronto, Toronto, ON, Canada; 9 Interdepartmental Division of Critical Care, Temerty School of Medicine, University of Toronto, Toronto, Canada; 10 Sunnybrook Health Sciences Centre, Toronto, ON, Canada; 11 Department of Medicine, Division of Critical Care Medicine, Université de Sherbrooke, Sherbrooke, QC, Canada; 12 Department of Medicine, Sinai Health and Interdepartmental Division of Critical Care Medicine, University of Toronto, Toronto, ON, Canada; Universidad de La Sabana, COLOMBIA

## Abstract

**Background and objective:**

Collection of biosamples for translational research studies is vital for understanding biological pathways, discovering disease-related biomarkers, and identifying novel therapeutic targets. However, a lack of infrastructure for sample procurement, processing, storage, and shipping may hinder the ability of clinical research units to effectively engage in translational research. The purpose of this study was to identify the barriers to biosampling-based translational research in the critical care setting in Canada.

**Methods:**

We administered an online survey to members of the Canadian Critical Care Trials Group (CCCTG), the Canadian Critical Care Translational Biology Group (CCCTBG), and the Canadian Critical Care Research Coordinators Group (CCCRCG). The survey focused on participants’ personal experience of biosampling research, research infrastructure, motivating factors, and perceived barriers.

**Results:**

We received 59 responses from 31 sites, including 6 community intensive care unit (ICU) sites. The overall response rate was 11.3%. The majority of respondents were research coordinators (44%), followed by clinician-investigators (33.8%), graduate students (10.2%), and PhD-investigators (8.5%). Although most (63.8%) respondents reported an interest in participating in translational research, they also reported that their ICUs were currently contributing to a third of the number of translational studies compared to clinical studies. For respondents with experience in participating in translational research studies, the most common barriers were lack of funding, lack of time, and insufficient research staff. For respondents without previous experience, the perceived facilitators were more interest from their research group, improved training/mentorship, increased funding, and better access to laboratory equipment.

**Conclusions:**

Our survey found that the majority of participants were interested in and recognize the value of participating in biosampling-based translational research but lacked funding, time, and research personnel trained in biosampling protocols. Our survey also identified factors that might encourage participation at new sites. Addressing these barriers will be a key step towards increasing translational research capacity across Canada.

## Introduction

Translational research is characterized as a two-way bridge between discoveries in the lab and applications that can improve health outcomes for patients [1]. Effective translational research requires a multidisciplinary team of basic science researchers, clinicians, research coordinators, lab staff, and biostatisticians to design and conduct research studies that produce meaningful results with clinical application [2]. Effective translational research should also represent the full diversity of the patient population, including patients of different ethnicities, cultural backgrounds and socio-economic status [2]. This can be achieved through consideration of diversity in geography and type of intensive care units (ICUs) in both academic and community hospitals across Canada.

A critical component of translational research is the collection of high-quality biological specimens (such as blood, tissues, or urine) from patients, either in the context of observational studies or randomized clinical trials. Biospecimen repositories (i.e. biobanks) are essential for the investigation of biological pathways, the validation of disease-related biomarkers, and the identification of novel therapeutic targets [3]. However, a lack of experience and infrastructure for sample collection, handling, processing, storage, and transport may hinder the ability of research groups to engage in translational research projects. This may be especially true in community hospitals which typically lack basic infrastructure and research personnel to engage in research [4].

The purpose of this study was to gain insight into the current translational research capacity, and well as barriers and enablers to biosampling-based translational research in the critical care setting in Canada. We surveyed members of the Canadian Critical Care Translational Biology Group (CCCTBG), the Canadian Critical Care Trials Group (CCCTG), and the Canadian Critical Care Research Coordinators Group (CCCRCG). Together, these groups comprise one of the largest critical-care focused forums in Canada, represented by over 500 members from multiple disciplines, including healthcare professionals, basic science researchers, and research staff and trainees. The groups are investigator-led and peer-peer funded, with a focus to improve the care of critically-ill patients through investigator-led research. Our internet-based survey focused on participants’ personal experience with biosampling-based research, availability of biosampling infrastructure, level of interest, motivation factors, and perceived barriers. The goal was to identify solutions that will improve translational research capacity within critical care in Canada.

## Materials and methods

### Survey development

Research ethics approval for this online survey was obtained from the Hamilton Integrated Research Ethics Board (HiREB #15464). No identifying information was collected. In the survey, the terms “translational research” was defined as biosampling-based translational research within human observational or interventional studies. The survey was developed using the guide published by Burns et al. [5]. The pilot survey was developed in English by EC and PL and assessed by JT, AB, CD, RF, FL, and SM for face validity and internal consistency. Revisions suggested from this assessment were completed by EC and PL. The online survey was developed using Google Forms. Multiple question formats were used, including multiple choice questions, yes/no questions, Likert scale questions, and open-ended responses. The final draft of the survey was pre-tested by 2 faculty members as well as 5 trainee members of the CCCTG and CCCTBG prior to administration. The final survey had questions which were grouped into multiple categories: respondent demographics (4 questions), previous experience in biosampling-based research (4 questions), barrier ratings (8–10 questions depending on which set of questions were shown), local research infrastructure (4 questions), previous research conducted by the lab (1 question), and previous clinical/translational study details (9 questions). Generally, respondents were asked to answer questions based on their personal perspectives. The length of the survey was approximately 5–10 minutes.

### Survey administration

The survey was administered between March 16, 2023 and April 25, 2023. Participants included clinician investigators (MD or MD/PhD), non-clinical investigators (PhD), graduate students, research coordinators, post-doctoral fellows, and research staff (e.g., lab technicians, clinical staff). Participants were not required to have previous translational research experience and the set of available questions was customized according to translational research experience. The survey link was sent via email to 521 members of the CCCTG, CCCTBG, and CCCRCG, with a reminder email sent to survey recipients four weeks later. These groups were chosen as they represent a sizeable portion of the Canadian critical care community, and are closely involved in member research (offering two annual meetings to discuss ongoing projects as well as manuscript review, project funding, and other services). To contact potential participants, administrative staff of the CCCTG, CCCTBG, and CCCRCG were asked to send an email containing survey details to members of the three groups on the researchers’ behalf. Participants were informed at the start of the survey that by submitting the completed survey, they consent to take part in the study.

### Statistical analysis

All results were summarized in Microsoft Excel and graphed using GraphPad Prism. Continuous variables were summarized as mean and standard deviation or median and interquartile range, as appropriate.

## Results

### Participant demographics

**Fig 1:**
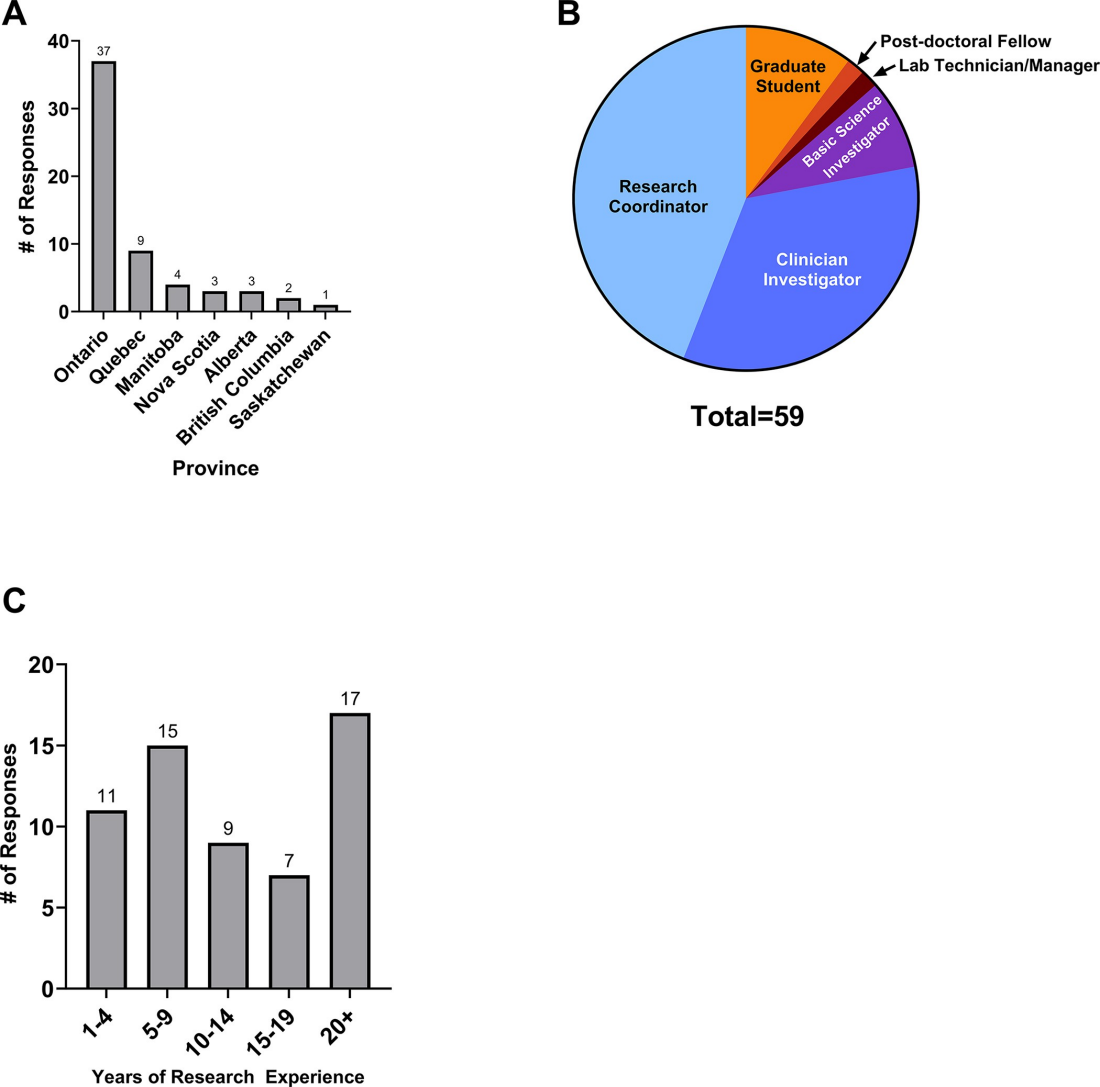
Participant demographics. (A) Number of responses from each province. (B) Participants’ professions. (C) Research experience of participants (in years).

The online survey was sent to 521 individuals with an overall response rate of 11.3%. The 59 responses were received from participants in 7 provinces and 31 sites across Canada, including 6 community sites (Fig 1A). Of the respondents, 51 were from academic sites while 8 were from community sites. Geographically, 37 (62.7%) of the respondents are located in Ontario, followed by 9 (15.3%) in Quebec, 4 (6.8%) in Manitoba, 3 (5%) each in Nova Scotia and Alberta, 2 (3.4%) in British Columbia, and 1 (1.7%) in Saskatchewan. The majority of the respondents were research coordinators (44%), followed by clinician investigators (33.8%), graduate students (10.2%), and PhD-level investigators (8.5%) (Fig 1B). 67.2% of respondents identified as women; 68% of participants identified as White, 10% as South Asian, 6.7% as East Asian, 5% identified as Black, 5% as Southeast Asian, 1.7% as Indigenous, and 1.7% as Middle Eastern (S1 Table). Over half (56%) of participants had more than 10 years of experience in lab-based or clinical research, and 28.8% of participants had over 20 years of experience (Fig 1C).

### Prior experience in translational research

**Fig 2:**
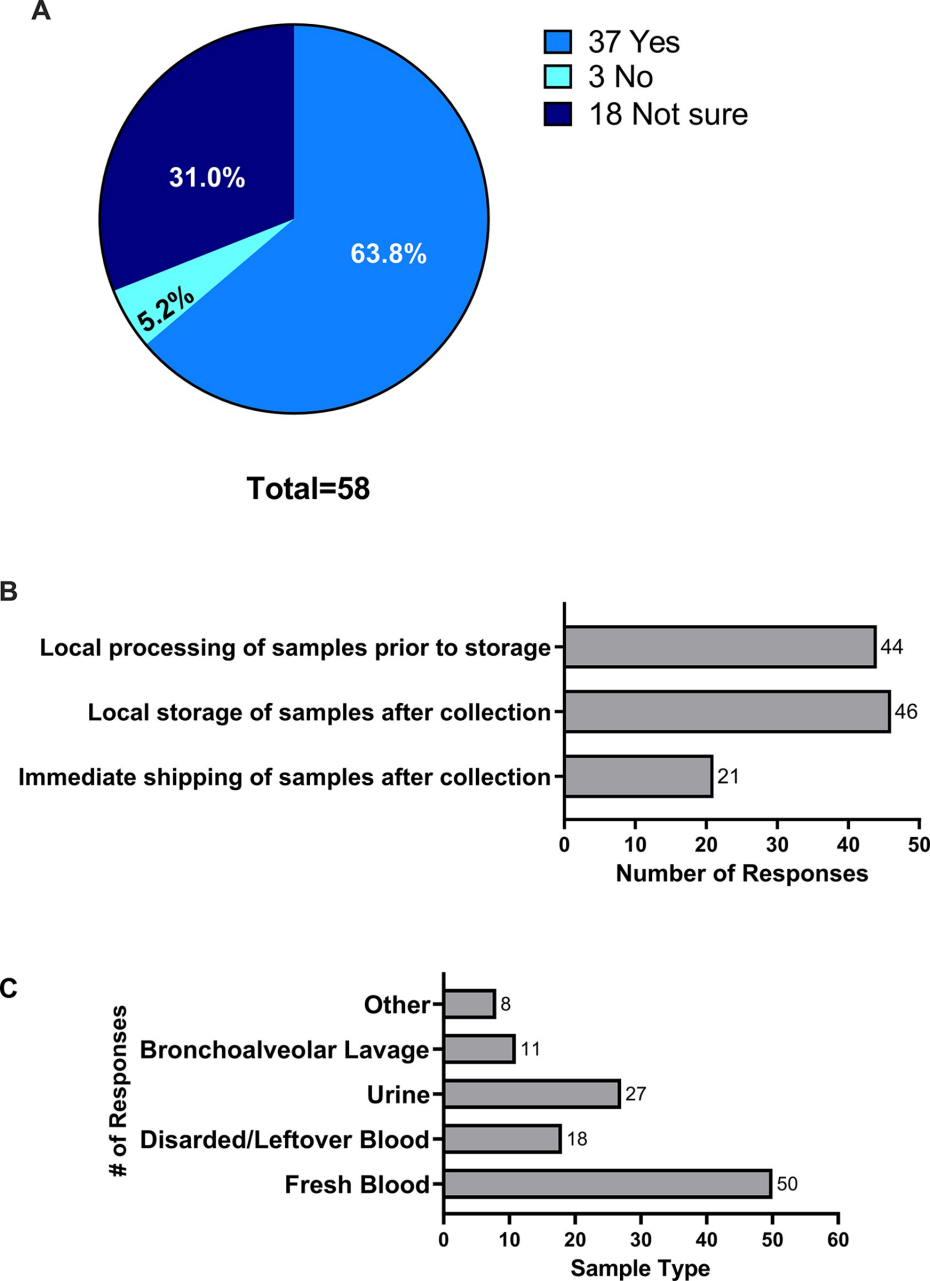
Experience and interest in translational research. (A) Percentage of participants interested in conducting translational research in the future. (B) Type of sample processing conducted in previous studies. (C) Biological sample types collected in previous studies.

With respect to translational research (which we defined as biosampling-based translational research), 48 (81.4%) participants stated that their research group had conducted translational research in the past. When asked about interest in conducting translational research in the future, 63.8% of participants stated “yes,” 5.2% stated “no,” and 31% stated that they were unsure (Fig 2A). In the 11 respondents who had not previously participated in translational research, 6 (60%) stated that their research groups were interested in conducting translational research in the future, 4 (40%) stated that they were unsure, and 1 participant did not respond.

### Types of specimens collected

Regarding the types of specimens collected, 50 participants responded that the most common types of samples collected were fresh blood (100%), urine (54%), discarded/leftover blood (36%), and bronchoalveolar lavage fluid (22%). 8 (16%) participants reported that they had also collected other types of samples (Fig 2B). With respect to local processing, 14 (42.8%) participants had conducted at least one study in which samples were shipped immediately after collection, 46 (93.9%) had conducted a study in which samples were stored locally after collection, and 44 (89.8%) had conducted a study in which samples were processed and stored locally after collection (Fig 2C).

### Access to research infrastructure for translational research

**Fig 3:**
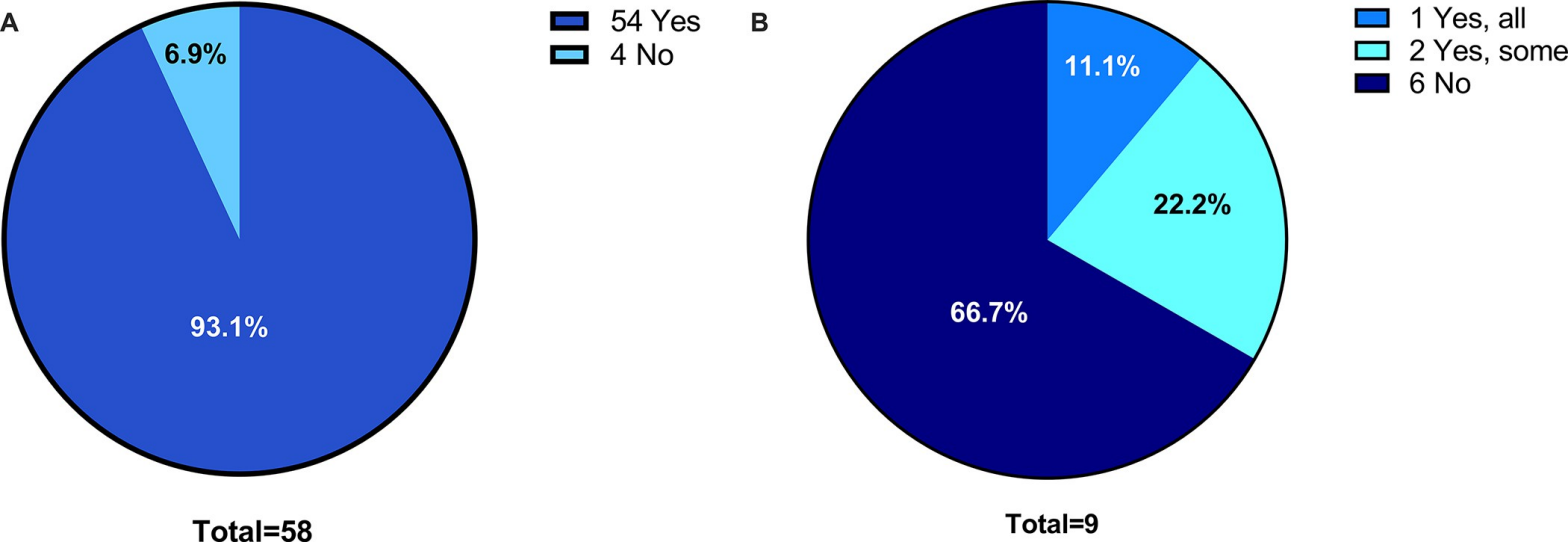
Access to essential laboratory equipment for biosampling research. (A) Access to a tabletop centrifuge, a 4°C fridge, and a -20°C freezer. (B) Participants who answered “no” to the question in Fig 5A were asked if their current funding allowed their group to purchase the listed equipment if necessary.

Participants were asked about the availability of biosampling infrastructure at their sites, including details on laboratory equipment and research staff. 54 (93.1%) participants stated that they had access to a laboratory with a functional tabletop centrifuge, a 4°C fridge, and a -20°C freezer (Fig 3A). This included laboratories owned by research groups as well as by a third-party. Of the 4 (6.9%) participants who did not have access to a laboratory, only one participant indicated that their research group could procure the missing equipment with their current funding (Fig 3B). Of the 8 responses from community sites, all stated that they had access to laboratory equipment, but only 2 (25%) stated that they owned the equipment. In addition, participants were asked to indicate which lab-related items they require but are unable to procure. The most common items were freezer space, lab bench/biosafety cabinet space, and sample shipment equipment (e.g. dry ice, shipping boxes).

**Fig 4:**
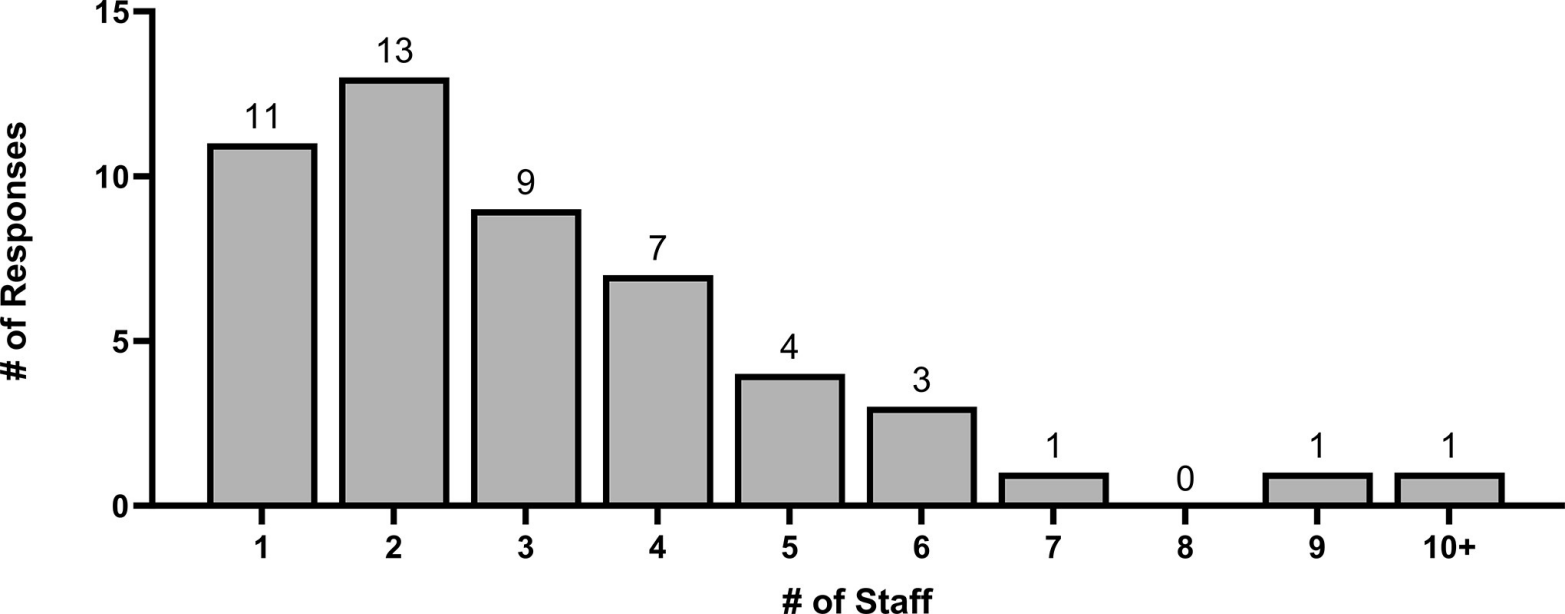
Number of full-time equivalent research staff (including research coordinators and research assistants) available in the ICU.

**Fig 5:**
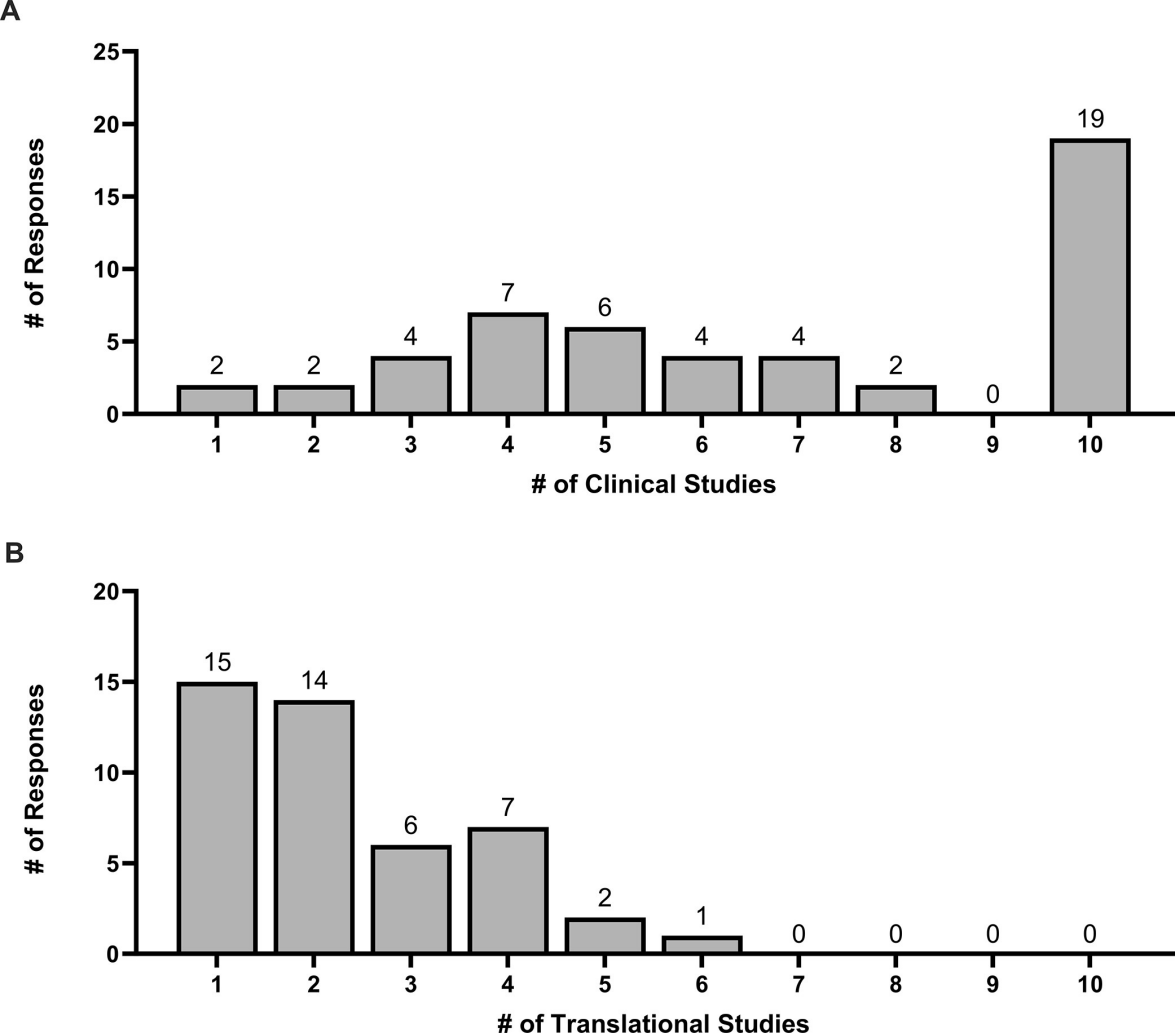
Number of studies being conducted in participant ICUs at the time of the survey. (A) Number of clinical studies. (B) Number of translational studies.

Regarding research staff, 33 (66%) participants stated that their ICU research group comprises fewer than 4 full-time equivalent research staff (including research coordinators and research assistants) (Fig 4). When stratified by type of site, 7 (87.5%) respondents from community hospitals stated that they had fewer than 4 full-time equivalent research staff. Furthermore, 25 (50%) participants stated that their ICUs were currently running ≥ 6 clinical studies while 29 (64.4%) participants were running ≤ 2 translational studies (Fig 5). For respondents from community hospitals, 7 (87.5%) stated that their ICUs were running ≤ 6 clinical studies and ≤ 2 translational studies. For respondents from academic hospitals, 27 (52.9%) stated that their ICUs were running ≥ 6 clinical studies and 36 (70.6%) stated that their ICUs were running ≤ 2 translational studies.

### Barriers to participation in translational research

**Fig 6:**
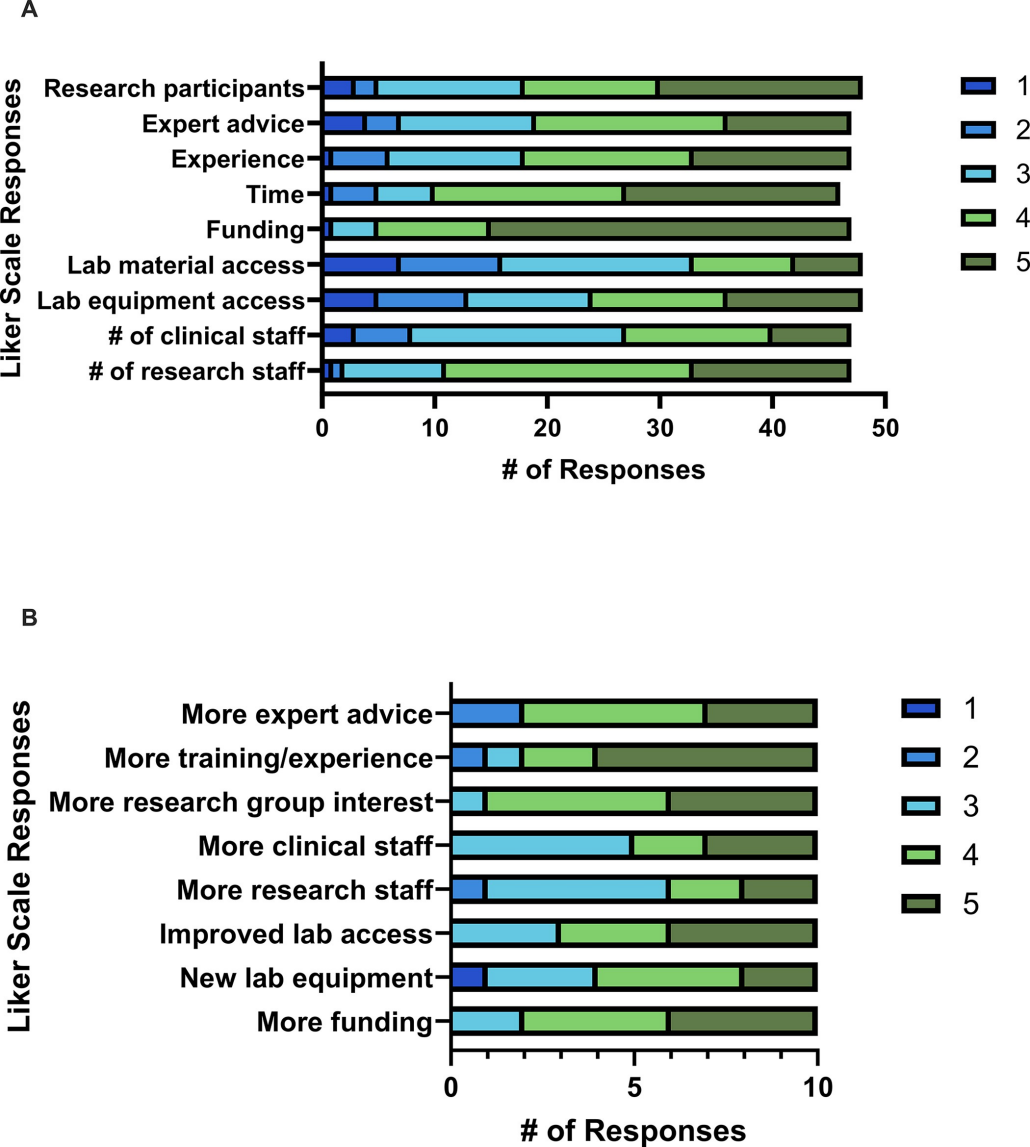
Barriers and facilitators of translational research participation. (A) Perceived impact of potential facilitators amongst participants with experience conducting translational research. (B) Perceived impact of potential facilitators amongst participants with no experience conducting clinical research.

Survey participants were asked to rate the importance of perceived barriers to translational research. Participants rated their responses on a 5-point Likert scale, ranging from 1 (strongly disagree) to 5 (strongly agree). Amongst participants with prior translational research experience, 85.4% stated that they had faced barriers to conducting translational research. The most common barriers were a lack of funding (4.5, SD = 0.8), lack of time (4.1, SD = 1.0), and insufficient research staff/trainees (4.0, SD = 0.9) (Fig 6A).

Amongst participants who did not have prior experience in translational research, factors that would increase engagement in future translational research were: “More interest from research group” (4.3, SD = 0.7), “More training/experience in how to conduct biosampling-based translational research” (4.3, SD = 1.1), “More funding for translational research” (4.2, SD = 0.8), and “Improved access to a physical laboratory” (4.1, SD = 0.9) (Fig 6B).

To determine whether sample processing was a major barrier to participating in translational research, participants were asked if their ICU had ever declined participating in a clinical study in which biosampling was required for participation; 7 (13.5%) responded “yes” and 24 (46.2%) responded “no,” with the remainder stating that they were unsure. In addition, when asked if their ICUs had ever declined participating in optional biological sample collection for a clinical study, 17 (32.7%) answered “yes”, 20 (38.5%) answered “no”, and the remainder were unsure.

### Other barriers

Participants were asked to describe any to describe any additional barriers faced in conducting translational research studies which had not been covered by the questions in the survey. Of the 17 responses, the most common barriers listed were insufficient equipment availability and/or staff availability (7 responses), administrative delays (such as fulfilling regulatory requirements and obtaining ethics approval) (6 responses), and third-party costs and/or lack of funding (6 responses).

## Discussion

In this survey study, Canadian critical care researchers were asked to report on their experience in conducting translational research studies as well as the barriers faced in conducting these types of studies. While most participants reported being interested in ICU-based translational research, there are currently 3-times fewer translational studies compared with clinical studies, representing a notable gap in translational ICU research in Canada. Amongst participants with prior translational research experience, the most common barriers to participation included insufficient financial support, time, and personnel. Amongst participants without prior experience, the most common perceived facilitators were more interest from their peers, improved training/experience, increased funding, and better access to laboratory equipment. To our knowledge, this is the first study which describes the translational research landscape in academic and community critical care sites across Canada. Our findings highlight the need and potential avenues to increase translational research capacity in Canada.

In the United States, translational research is supported by programs funded by the National Institutes of Health (NIH) such as the Clinical and Translational Science Award (CTSA) hubs (https://ncats.nih.gov/ctsa/about/hubs). The CTSA hubs are a national network of medical research institutions with the common goal of accelerating the translation of scientific discoveries to improve patient care. In response to the COVID-19 pandemic, the CTSA hubs took active steps to build high-quality biorepositories which required a shift in biorepository models from investigator-initiated to institution-supported [3]. Establishing infrastructure at the institutional level was shown to reduce start-up costs associated with activities such as applying for ethics approval, preparing study protocols, and training research staff. Many CTSA hubs also organized governance committees to provide transparency and fair access to specimens, as well as to identify important targeted populations such as vulnerable groups [3].

A key lesson learned from the CTSA COVID biorepositories is the importance of including diverse populations of patients in order to support high-quality research that is generalizable [3]. Institutions can explore ways to increase enrollment success such as video-based or e-Consent strategies [3]. In Canada, the majority of ICU care is provided by community hospitals, which have limited participation in clinical trials or other research [6, 7]. Given that community sites disproportionately serve racialized and socio-economically deprived Canadians, their involvement in research is critical to improve research participant diversity in Canadian critical care trials [6]. A recent Canadian study demonstrated that community ICUs can conduct research with comparable recruitment rates, consent rates, and protocol adherence when compared to academic ICUs [6].

With respect to initiatives that support critical care translational research in Canada, the CCCTBG has been at the national forefront in developing and providing standard operating procedures (SOPs), instructional videos, webinars, and workshops on the collection, handling, processing, and storage of biospecimens from critically ill patients (https://www.ccctg.ca/our-initiatives/canadian-critical-care-translational-biology-group). To date, the CCCTBG has supported over 20 investigator-initiated translational research programs that focus on disease pathophysiology, biomarker discovery, and early phase clinical trials. Another initiative is the COVID-19 Network of Clinical Trials Networks (Network of Networks) that was established in 2021 by the CCCTG to create a durable infrastructure that embeds research into clinical care. The Network of Networks is a pan-Canadian partnership of clinical trials networks with the goal of strengthening and supporting new and existing trials (https://www.ccctg.ca/our-initiatives/network-of-networks). Included in the Network of Networks are CCCTBG and CCCTG members who are leading Working Groups to enhance translational research capacity at new and established sites through (a) increasing training and mentorship, (b) tracking details of clinical trials including those with biobanking components, (c) sharing research infrastructure, and (d) fostering collaborations between study investigators and community ICUs.

A limitation of this study is that the overall response rate was only 11.3% which may reflect our ability to only recruit participants via email (rather than in-person or by phone). The low response rate potentially limits the validity of the data due to an increased risk of response bias. However, the respondents represented diverse sites, educational backgrounds, research commitments, and clinical responsibilities. In addition to surveying the CCCTBG, which has a strong focus on translational research, we also surveyed members of the CCCTG and CCCRCG, who may have less exposure and experience in translational research. Since the participants were members of the CCCTG, CCCTBG, and CCCRCG, they were likely to have already been part of research collaboratives or employed in research-intensive settings (such as academic hospitals). This may have produced a bias towards participants who had previously taken part in research, as exemplified by the participation of members from only 6 community hospitals. The total number of teaching and non-teaching hospitals in Canada is summarized in S1 Table. Finally, participants from Ontario (63%) were overrepresented relative to those from other provinces. However, Ontario is the most populous province, representing 38.5% of the Canadian population and has several research-active community hospital ICUs.

## Conclusions

Our survey found that Canadian researchers are interested in participating in translational research in the critical care setting, but face significant barriers including insufficient funding, time, and research personnel. Increasing participation by engaging new sites will require strategies to enhance mentorship, training, funding, and access to laboratory facilities. Our study highlights the importance of developing research models that can sustain current and new research groups with translational research capacity, particularly those from community ICU sites.

## Supporting information

S1 Table Number of teaching and non-teaching hospitals in Canada by province. (DOCX)

S2 Table Population groups with which participants identified. (DOCX)

S3 Table Gender of the participants. (DOCX)
