# Supplementary material for: Barriers to participation in biosampling-based translational research: A cross-sectional survey of Canadian critical care researchers
Source: PLoS One. 2024 May 17;19(5):e0303304. doi: 10.1371/journal.pone.0303304 (PMC11101101; doi:10.1371/journal.pone.0303304)
Supplement: S1 Table — (DOCX) [file pone.0303304.s001.docx]

**Supporting information**

**S1 Table. Number of Teaching and Non-Teaching Hospitals in Canada by Province**

| **Province** | **Number of Teaching Hospitals** | **Number of Non-Teaching Hospitals** |
| --- | --- | --- |
| **Alberta** | 8 | 94 |
| **British Columbia** | 12 | 74 |
| **Manitoba** | 2 | 70 |
| **New Brunswick** | 1 | 24 |
| **Newfoundland and Labrador** | 1 | 33 |
| **Nova Scotia** | 2 | 35 |
| **Nunavut** | 0 | 4 |
| **Ontario** | 17 | 123 |
| **Prince Edward Island** | 0 | 7 |
| **Quebec** | 10 | 23 |
| **Saskatchewan** | 5 | 56 |
| **Yukon** | 0 | 3 |
| **Total (Canada)*** | 58 | 546 |

Data from Canadian Institutes for Health Research: Hospital Beds Staffed and In Operation, 2021-2022. Data for Northwest Territories unavailable.
