# Supplementary material for: Barriers to participation in biosampling-based translational research: A cross-sectional survey of Canadian critical care researchers
Source: PLoS One. 2024 May 17;19(5):e0303304. doi: 10.1371/journal.pone.0303304 (PMC11101101; doi:10.1371/journal.pone.0303304)
Supplement: S2 Table — (DOCX) [file pone.0303304.s002.docx]

**S2 Table: Population groups with which participants identified**

| **Population Group(s)** | **# of Responses** | **% of Total** |
| --- | --- | --- |
| Black (e.g., African, Afro-Caribbean, African Canadian Descent) | 3 | 5 |
| East Asian (e.g., Chinese, Korean, Japanese, Taiwanese descent) | 4 | 6.7 |
| Indigenous (First Nations, Inuk/Inuit, Métis) (e.g., First Nations, Inuk/Inuit, Métis descent) | 1 | 1.7 |
| Latino American (Hispanic or Latin American descent) | 0 | 0 |
| Middle Eastern (e.g., Arab, Persian, West Asian descent (e.g., Afghan, Egyptian, Iranian, Kurdish, Lebanese, Turkish)) | 1 | 1.7 |
| South Asian (e.g., South Asian descent (e.g., Bangladeshi, Indian, Indo-Caribbean, Pakistani, Sri Lankan)) | 6 | 10 |
| Southeast Asian (Cambodian, Filipino, Indonesian, Thai, Vietnamese, other Southeast Asian descent) | 3 | 5 |
| White (e.g., European descent) | 41 | 68.3 |
| Prefer not to answer | 1 | 1.7 |
| **Total*** | 60 |  |

*Responses where more than one option were selected were listed as separate entries.
