# Supplementary material for: Barriers to participation in biosampling-based translational research: A cross-sectional survey of Canadian critical care researchers
Source: PLoS One. 2024 May 17;19(5):e0303304. doi: 10.1371/journal.pone.0303304 (PMC11101101; doi:10.1371/journal.pone.0303304)
Supplement: S3 Table — (DOCX) [file pone.0303304.s003.docx]

**S3 Table: Gender of the Participants**

| **Gender** | **# of Responses** | **% of Total** |
| --- | --- | --- |
| Woman | 39 | 67.2% |
| Man | 18 | 31.0% |
| Transgender | 0 | 0 |
| Non-binary/non-conforming | 0 | 0 |
| Prefer not to say | 1 | 1.7% |
| Total | 58 |  |
